# A Diagnostic Pitfall: Secondary Syphilis Mimicking Psoriasis in a Patient With Alcoholic Liver Disease

**DOI:** 10.7759/cureus.104720

**Published:** 2026-03-05

**Authors:** Julia L Armstrong, Erica Rankin, Prakriti Singh Shrestha, Samia S Siddiqui, Arifa Turkistani, Jorge E Evora, Inima Rodriguez Caballero, Arohi Ghandi-Patel, Vanessa S Samaniego, Ivan D Rodriguez

**Affiliations:** 1 Medicine, Nova Southeastern University Dr. Kiran C. Patel College of Osteopathic Medicine (KPCOM), Fort Lauderdale, USA; 2 Family Medicine, Division of Research and Academic Affairs, Larkin Community Hospital, Miami, USA; 3 Family Medicine, Larkin Community Hospital, Miami, USA; 4 Internal Medicine/Family Medicine, Larkin Community Hospital, Miami, USA

**Keywords:** alcoholic dermatoses, alcoholic liver cirrhosis, atypical secondary syphilis, psoriasis, psoriasis variant, syphilis screening

## Abstract

Syphilis is a sexually and vertically transmitted infection caused by *Treponema pallidum* that remains a significant public health concern due to its rising incidence and protean clinical manifestations. The disease is characterized by distinct stages and has the potential to involve multiple organ systems, leading to serious complications if diagnosis and treatment are delayed. Secondary syphilis, in particular, is notorious for its wide spectrum of clinical presentations and its ability to closely mimic numerous dermatologic and systemic conditions, often resulting in diagnostic uncertainty.

We present a case of secondary syphilis in a 52-year-old male patient with a known history of chronic psoriasis, whose diffuse papular eruption closely resembled a psoriasiform flare. The clinical picture was further complicated by underlying alcoholic cirrhosis with associated jaundice, which obscured classic cutaneous and systemic features typically associated with secondary syphilis. Initial laboratory evaluation yielded indeterminate serologic findings, contributing to diagnostic delay and highlighting the limitations of relying on a single test in complex clinical scenarios. The coexistence of a preexisting inflammatory skin disorder and chronic liver disease significantly lowered clinical suspicion for syphilis, as the patient’s dermatologic findings were initially attributed to an exacerbation of psoriasis. Ultimately, a thorough clinical reassessment, repeat serologic testing, and careful consideration of alternative diagnoses led to the identification of secondary syphilis. This case underscores the diagnostic challenges posed by syphilis in patients with comorbid dermatologic and systemic conditions and illustrates the importance of maintaining a high index of suspicion, even when cutaneous findings appear consistent with an established underlying disease. It also emphasizes the need for routine syphilis screening in at-risk populations and in patients with atypical or refractory skin manifestations. Early recognition and appropriate treatment are critical to preventing disease progression and long-term complications, reinforcing the role of vigilant clinical evaluation in modern medical practice.

## Introduction

Syphilis is a bacterial infection caused by the spirochete* Treponema pallidum (T. pallidum),* which spreads through sexual contact or vertical transmission [1]. Despite the availability of effective treatment, syphilis has re-emerged as a major public health issue worldwide [2]. Recent epidemiologic data from the United States (US) demonstrate a marked rise in incidence, underscoring the ongoing challenges in prevention, screening, and early detection [2]. In 2022, an estimated eight million cases were reported globally, reflecting a substantial increase over recent years [3]. In the United States, a 9.3% increase in cases was reported from 2021 to 2022 [2]. Prompt recognition and treatment are essential, as untreated syphilis can progress to advanced stages with potentially devastating cardiovascular, neurologic, and sensory sequelae [2].

Syphilis is often referred to as “*the great imitator*” due to its highly variable clinical manifestations, which differ by disease stage and frequently resemble other dermatologic or systemic disorders, such as psoriasis in this case, particularly atypical or guttate forms [4,5]. This case highlights the diagnostic challenges posed by overlapping clinical features and emphasizes the importance of maintaining a high index of suspicion for syphilis, even when skin findings appear consistent with an established underlying condition and indeterminate initial labs.

## Case presentation

A 52-year-old male with a past medical history of hypertension, chronic alcohol use, tobacco use, and psoriasis presented to our hospital with a one-month history of progressive weakness and dermatologic symptoms. The patient reported chronic alcohol consumption of approximately eight ounces of beer daily for most of his adult life and a 34-pack-year history of tobacco use. He presented with complaints of generalized weakness, hand pain and pallor reportedly noticed by coworkers.

The patient’s dermatologic history was significant for psoriasis since the age of 17 years, confirmed by skin biopsy in 2007 while incarcerated. He reported poor adherence to outpatient dermatology follow-up and intermittent exacerbations historically responsive to topical corticosteroids. His most recent severe flare occurred in 2022 and was treated with weekly methotrexate and topical hydrocortisone, along with intramuscular triamcinolone, with good clinical response. However, since this treatment was received outside the United States, there was no proper documentation available regarding dosing or frequency.

Three weeks prior to presentation to the emergency department, he noted the development of erythematous, papular, non-pruritic lesions over the trunk, palms of both hands, and soles of both feet, accompanied by mild bilateral pedal edema. Three days prior to admission, he experienced worsening swelling, exertional dyspnea, yellow discoloration of stools, and dark yellow urine.

Although he was unable to estimate the number of lifetime sexual partners, he reported no sexual activity in the past four to five years. His HIV status was negative. He endorsed a remote history of syphilis more than 35 years ago; however, documentation regarding prior treatment and dosing was unavailable. The patient had no established primary care provider, and his last physician visit was approximately 15 years prior to presentation. Due to the lack of available serologic and treatment records, both reinfection and previously untreated latent syphilis were considered. However, the presence of active cutaneous findings together with the patient's serologic profile was most consistent with secondary syphilis.

**Figure 1:**
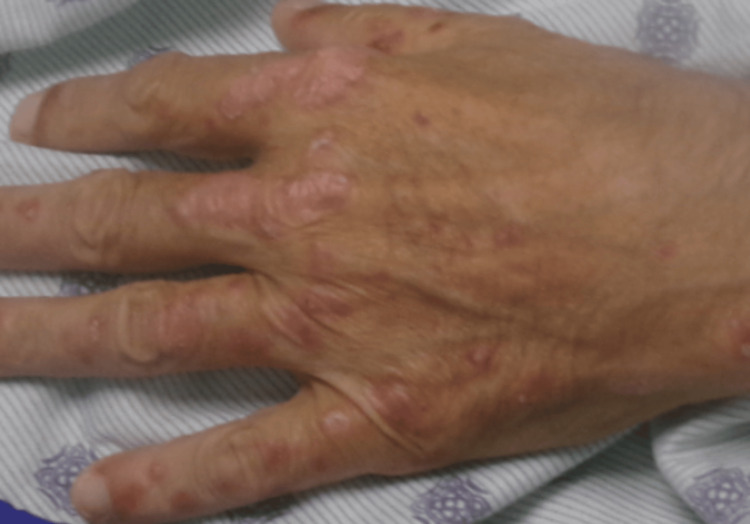
Scaly papular lesions involving the dorsal aspect of the hand.

On admission, the patient was alert and oriented, with no acute distress, though he appeared fatigued. Physical examination revealed scleral icterus and generalized jaundice. Vital signs were within normal limits. He endorsed systemic symptoms including weakness, exertional dyspnea, yellow stools, and abdominal distention. Neurologic examination demonstrated cranial nerves II-XII grossly intact. Dermatologic examination revealed scaly, red-purple papular lesions involving both hands and the soles of the feet with associated bilateral pedal edema (Figure 1).

**Figure 2:**
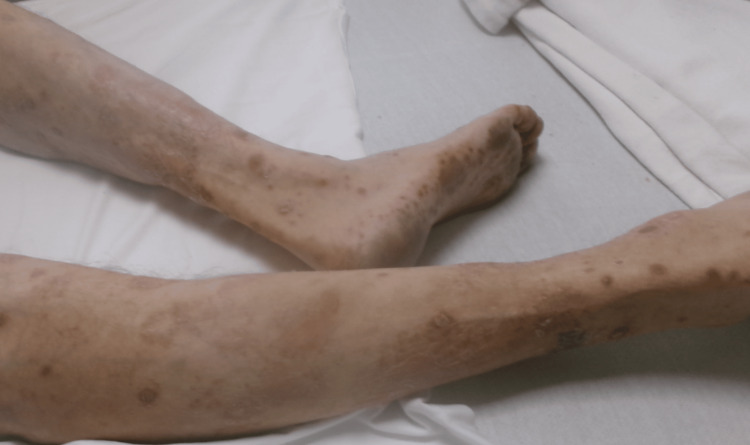
Scaly papular lesions on the lower extremities.

On further examination, the patient also had a rash affecting his lower extremities bilaterally (Figure 2). Admission laboratory studies revealed an increased serum ethanol level and a cholestatic pattern of liver injury, including elevated transaminases and cholestatic markers (Table 1).

**Table 1:** Admission laboratory values.

Parameters	Values	Reference range
Serum ethanol	34 mg/dL	0-10 mg/dL
Aspartate aminotransferase (AST)	302 U/L	15-46 U/L
Alanine aminotransferase (ALT)	125 U/L	0-35 U/L
Alkaline phosphatase (ALP)	528 U/L	38-126 U/L
Total bilirubin	9 mg/dL	0.2-1.3 mg/dL
Gamma-glutamyl transferase (GGT)	>2800 U/L	15-73 U/L

Given the patient’s long-standing alcohol use, scleral icterus, jaundice, and aspartate aminotransferase (AST) predominance over alanine aminotransferase (ALT), alcoholic hepatitis and cirrhosis-related dermatoses were initially considered the leading diagnoses. The differential diagnosis was broadened to include secondary syphilis and psoriasis.

**Figure 3:**
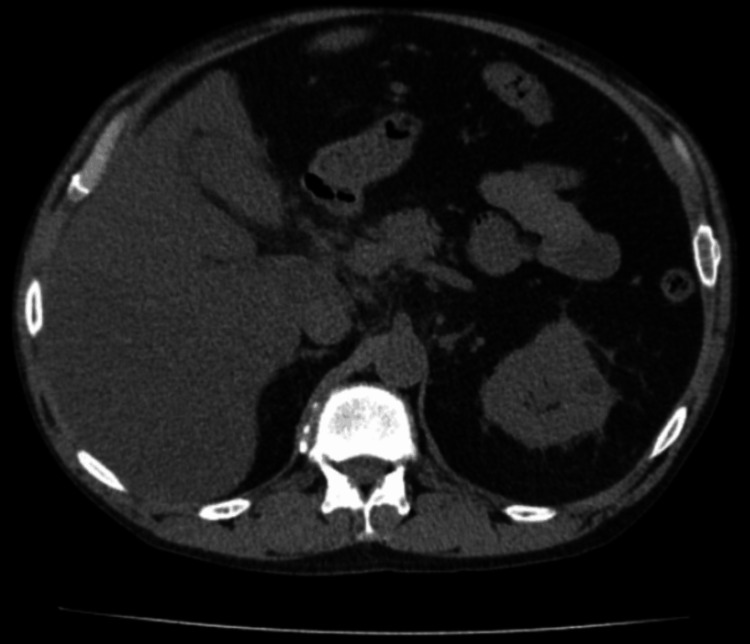
Non-contrast CT of the abdomen and pelvis revealing hepatic steatosis and misty mesentery in the RUQ. RUQ: right upper quadrant

A chest radiograph demonstrated no pulmonary consolidations or pleural effusions. Computed tomography (CT) of the abdomen and pelvis without contrast revealed hepatic steatosis and misty mesentery in the right upper quadrant, raising concern for possible sclerosing mesenteritis (Figure 3). No gallstones or findings suggestive of acute cholecystitis were identified.

**Figure 4:**
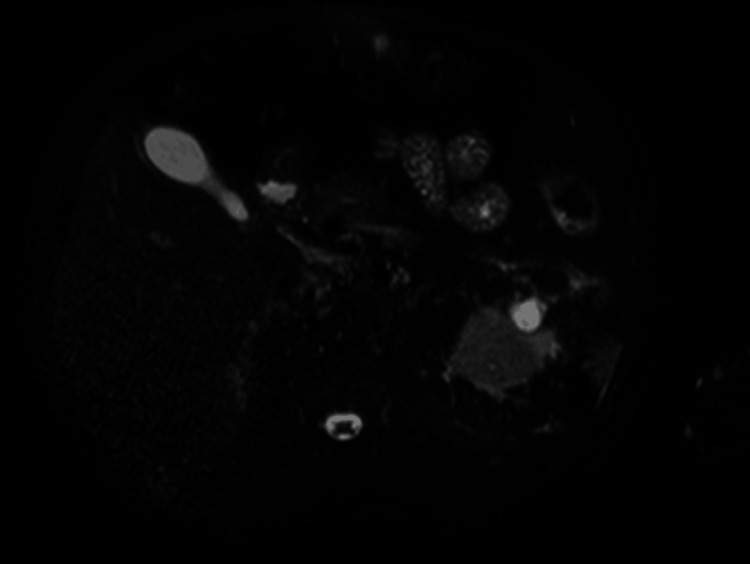
MRCP demonstrating indeterminate findings suggestive of possible acute acalculous cholecystitis. MRCP: magnetic resonance cholangiopancreatography

Gastroenterology was consulted and concluded that the patient’s elevated liver enzymes with a cholestatic pattern were most consistent with alcoholic hepatitis. Magnetic resonance cholangiopancreatography (MRCP) showed no evidence of choledocholithiasis or hepatic masses but demonstrated indeterminate findings suggestive of possible acute acalculous cholecystitis (Figure 4).

**Figure 5:**
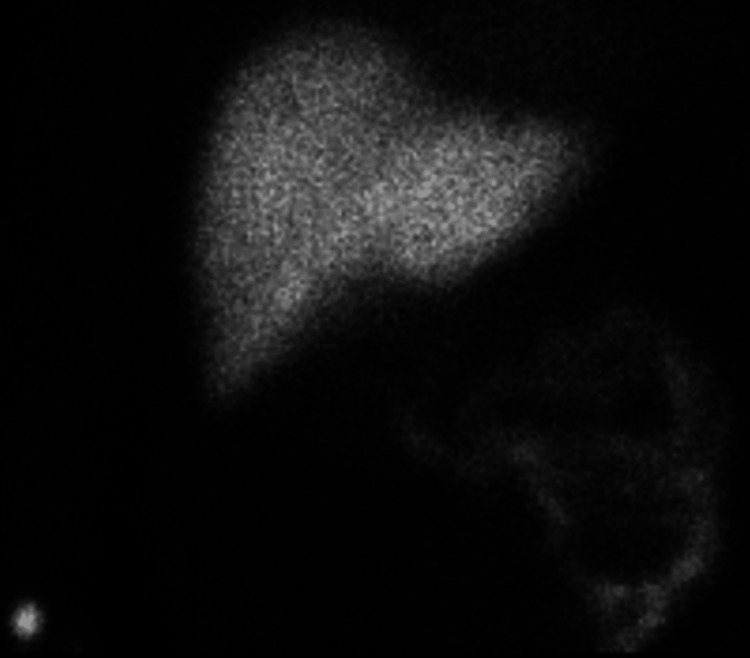
HIDA scan revealing partial and delayed gallbladder uptake. HIDA: hepatobiliary iminodiacetic acid

A hepatobiliary iminodiacetic acid (HIDA) scan revealed partial and delayed gallbladder uptake, suggestive of partial cystic duct obstruction, with differential considerations including local biliary dysmotility (Figure 5). The patient was strongly counseled on alcohol cessation and advised to undergo outpatient screening esophagogastroduodenoscopy (EGD) for evaluation of esophageal varices.

Dermatology and infectious disease services were consulted. An initial rapid plasma reagin (RPR) test was non-reactive; however, confirmatory fluorescent treponemal antibody absorption (FTA-ABS) testing returned reactive. Based on these findings, the patient was diagnosed with secondary syphilis in addition to alcoholic hepatitis.

During the current admission, the patient was started on triamcinolone acetonide 0.1% ointment applied twice daily to affected areas for psoriasis. Although the patient's clinical presentation was characteristic of secondary syphilis, due to uncertainty regarding prior treatment and infection history, he was treated with doxycycline 100 mg orally every 12 h for 28 days to cover syphilis of unknown duration.

Throughout hospitalization, the patient’s cutaneous lesions were controlled, and he was discharged with strict instructions to adhere to the prescribed medication regimen and to maintain close outpatient follow-up with dermatology, infectious disease, and gastroenterology specialists to ensure complete resolution of symptoms and ongoing management.

## Discussion

Syphilis remains a diagnostic challenge due to its diverse clinical manifestations and ability to mimic numerous dermatologic and systemic diseases, earning its designation as “the great imitator” [1,3,4]. This case highlights the complexity of diagnosing secondary syphilis in a patient with preexisting psoriasis and alcohol-related liver disease, where overlapping cutaneous findings and systemic symptoms obscured clinical suspicion.

In this case, long-standing psoriasis initially anchored the differential toward a psoriatic flare, particularly because the eruption involved the palms and soles, sites where palmoplantar psoriasis can present with erythematous, scaly lesions, and fissuring and may cause significant functional impairment [6]. Palmoplantar psoriasis closely resembles the psoriasiform eruptions of secondary syphilis, a diagnostic overlap well described in the literature [6,7]. In this case, several features were less typical for a routine psoriasis flare and supported a competing infectious etiology. The palmar/plantar lesions evolved from macules to papules over weeks, were described as non-pruritic, and became functionally limiting due to sensitivity and impaired grip, which is consistent with the high-impact disability associated with palmoplantar involvement [6,8]. The concurrent presence of widespread plaques, including genital involvement, created the appearance of a single unifying psoriasiform process, further lowering suspicion for infection [5,9]. Psoriasiform syphilis has been frequently mistaken for inflammatory dermatoses, resulting in delayed diagnosis and treatment [4,7,10]. This case reinforces that when rash morphology or distribution changes from a patient’s baseline psoriasis, particularly with new palm/sole disease, clinicians should broaden the differential and test for infectious mimics rather than attributing all findings to a flare [3,6,8,11].

Additionally, the patient’s systemic findings, including jaundice, scleral icterus, and markedly elevated liver enzymes, were initially attributed to alcoholic hepatitis and chronic liver disease. Alcohol-related liver disease is associated with common cutaneous findings (e.g., jaundice, pruritus, edema, palmar erythema, and other non-specific skin changes), which can distract from alternative diagnoses when a prominent non-infectious explanation is available [12,13]. Importantly, secondary syphilis can present with systemic manifestations (e.g., malaise, fatigue, lymphadenopathy, myalgias) in addition to mucocutaneous disease, and clinicians should avoid excluding sexually transmitted infections solely because a competing diagnosis appears to explain constitutional symptoms or laboratory abnormalities [1,3,4].

In this patient, the initial non-reactive rapid plasma reagin (RPR) test further complicated the diagnosis. False-negative nontreponemal tests can occur in secondary syphilis due to the prozone phenomenon (i.e., high antibody concentrations interfering with flocculation) or other host/test factors; when clinical suspicion remains high, repeat nontreponemal testing (including dilution to assess for prozone) and confirmatory treponemal testing should be pursued rather than accepting a single negative RPR as exclusionary [3,14-16]. Given the patient’s remote history of syphilis and chronic liver disease, confirmatory treponemal testing was essential and ultimately supported treatment in the setting of discordant initial screening results [3,15,16].

This case underscores the importance of maintaining a high index of suspicion for syphilis even in patients without reported recent sexual activity or with alternative explanations for dermatologic findings. Although the patient denied recent sexual exposure, his remote history of syphilis and lack of documented prior treatment raised concern for possible reinfection or inadequately treated disease. Current United States Preventive Services Task Force (USPSTF) screening recommendations emphasize testing in increased-risk populations; however, this case demonstrates that reliance on risk stratification alone may result in missed diagnoses when patients present with compatible clinical features [15,16]. Finally, from a public health perspective, the resurgence of syphilis represents a growing concern, and its capacity to masquerade as other conditions highlights the need for clinician awareness, timely confirmatory testing, and appropriate counseling to limit transmission and prevent late complications [2,3,17].

Psoriasis

Psoriasis is a common, chronic, immune-mediated inflammatory skin condition that encompasses a spectrum of clinical manifestations. Presentation can range from well-demarcated erythematous plaques with scale to more severe variants, including pustular and erythrodermic disease. The variability of psoriatic subtypes can pose challenges for prompt diagnosis and treatment and may require consideration of alternative pathologies when morphology, distribution, or symptom pattern deviates from baseline disease (Table 2). Diagnosis is typically clinical; biopsy may support the diagnosis in atypical cases or when mimics are strongly considered. 

**Table 2:** Overview of major psoriasis subtypes and their hallmark clinical features. This table was independently created by the authors of this study based on the information obtained from the National Psoriasis Foundation [9].

Types of psoriasis	Presentation
Plaque psoriasis	Red to silver-white scales
Guttate psoriasis	Small, round, red, or discolored spots
Inverse psoriasis	Inflamed, deep-red, or darkened smooth skin affecting skin folds
Pustular psoriasis	White, pus-filled bumps with erythematous border
Erythrodermic psoriasis	Discoloration with shedding of skin layers

Psoriasis commonly affects extensor surfaces of the upper and lower extremities, scalp, buttocks, and trunk, but it can involve any area, including the genital region. Palmoplantar psoriasis affects the palms and soles and can present as erythematous, scaly, thickened lesions that may be painful, fissured, and cause substantial functional impairment. This subtype has been repeatedly confused with psoriasiform syphilis, a form of secondary syphilis that may present with erythematous papulosquamous lesions on palms and soles [7]. In patients with known psoriasis, new palm/sole eruptions, particularly those that are non-pruritic, progressive, and associated with systemic symptoms, should prompt parallel evaluation for infectious mimics rather than escalation of psoriasis-directed therapy alone [3,7,10].

Syphilis

Syphilis presents in the following three stages: primary, secondary, and tertiary syphilis [1,3,15]. Primary syphilis classically presents with a painless chancre at the inoculation site, with an incubation period typically ranging from 10 to 90 days [1]. The lesion often resolves spontaneously within weeks, and because it may be painless or unnoticed, many patients do not seek care, contributing to missed diagnosis and later-stage presentation [1,3,4]. From a diagnostic standpoint, when a chancre is present, the most direct stage-appropriate laboratory confirmation is direct detection from lesion material (e.g., darkfield microscopy or other direct tests when available, including molecular methods), in addition to serologic testing with a nontreponemal test (RPR/Venereal Disease Research Laboratory {VDRL}) and a confirmatory treponemal assay (e.g., *Treponema pallidum*-particle agglutination {TP-PA}, enzyme immunoassay/chemiluminescent immunoassay {EIA/CIA}, fluorescent treponemal antibody absorption {FTA-ABS}) recognizing that very early primary infection may have negative serologies and may require repeat testing if suspicion remains high [15,16].

Secondary syphilis is characterized by a diffuse eruption that often includes the palms and soles and may appear macular, papular, or papulosquamous; mucous patches and condyloma lata may also occur [1,3,4,17]. Systemic manifestations can include fatigue, malaise, fever, myalgias, weight loss, and lymphadenopathy [1,3,4,17]. In this stage, recommended evaluation centers on serologic diagnosis using both a quantitative nontreponemal test (RPR/VDRL titer) and a treponemal test to confirm infection, with the nontreponemal titer serving as the baseline for treatment monitoring [15,16]. Because syphilis and HIV frequently co-occur and coinfection can affect presentation and follow-up considerations, patients diagnosed with syphilis should also receive HIV testing, and clinicians commonly assess for other STIs as clinically indicated [14,18].

Tertiary syphilis may occur years after infection and can involve multiple organ systems, including neurologic and cardiovascular complications [1,15,19]. Laboratory evaluation still includes syphilis serologies (nontreponemal and treponemal tests), but suspected late complications require targeted workup based on organ involvement - most importantly, if there are neurologic, ocular, or otologic symptoms, an evaluation for neurosyphilis/ocular syphilis/otosyphilis should include CSF analysis with CSF-VDRL (when positive, diagnostic), along with supportive CSF studies such as cell count and protein, interpreted in the clinical context [15,19].

From a diagnostic standpoint, syphilis remains challenging because serologies must be interpreted in a clinical context. Nontreponemal tests (RPR/VDRL) are useful for screening and treatment monitoring, but false-negative results can occur, including from the prozone phenomenon; discordant results warrant repeat testing with dilution and confirmatory treponemal assays rather than dismissal of the diagnosis [14-16].

Screening guidelines

The USPSTF guidelines recommend screening all high-risk individuals, including those with multiple sexual partners, HIV, men who have sex with men (MSM), sex workers, and incarcerated individuals, as well as all pregnant women during early pregnancy, due to the risk of fetal transmission [16]. The Traditional Algorithm (Forward Sequence) employs screening using a nontreponemal test (RPR/VDRL) followed by a confirmatory test (FTA-ABS, TP-PA, EIA/CIA) to diagnose individuals [16].

Treatment options and post-treatment monitoring

Treatment options according to CDC guidelines for primary, secondary, and early latent syphilis include benzathine penicillin G 2.4 million units IM × 1 dose or doxycycline 100 mg PO BID × 14 days as an alternative for those with a penicillin allergy [15]. Regarding late latent or tertiary syphilis, the CDC recommends benzathine penicillin G 2.4 million units IM weekly × 3 doses or doxycycline 100 mg PO BID × 28 days as an alternative treatment [15]. These guidelines are summarized in Table 3.

**Table 3:** CDC-recommended treatment regimens for syphilis by stage. This table was independently created by the authors based on information obtained from the Centers for Disease Control and Prevention (CDC) Sexually Transmitted Infections Treatment Guidelines, 2021 [15].

Population	Primary, secondary, and early latent	Late latent/tertiary (non-neuro)/unknown duration	Neurosyphilis
Adults/adolescents/pregnant (no penicillin allergy)	Benzathine penicillin G 2.4 million units IM × 1 dose	Benzathine penicillin G 2.4 million units IM weekly × 3 doses	Aqueous crystalline penicillin G 18-24 million units/day IV (3-4 MU q4h or continuous) for 10-14 days
Penicillin allergy (non-pregnant only)	Doxycycline 100 mg PO BID × 14 days or tetracycline 500 mg PO QID × 14 days or ceftriaxone 1 g IM/IV daily × 10-14 days (limited evidence)	Doxycycline 100 mg PO BID × 28 days or tetracycline 500 mg PO QID × 28 days or ceftriaxone 1 g IM/IV daily × 10-14 days (limited evidence)	Ceftriaxone 2 g IM/IV daily × 10-14 days (acceptable alternative)
Pregnant + penicillin allergy	Desensitization → benzathine penicillin G 2.4 million units IM × 1 dose	Desensitization → benzathine penicillin G 2.4 million units IM weekly × 3 doses	Desensitization → aqueous crystalline penicillin G 18-24 million units/day IV for 10-14 days

Because treponemal tests (FTA-ABS, TP-PA, EIA) remain positive for life, quantitative RPR/VDRL titers are used to monitor treatment. Regarding follow-up, RPR/VDRL titer levels are re-checked at six and 12 months for primary or secondary syphilis, and six, 12, and 24 months for latent syphilis [15].

Treatment failure/recurrence

While benzathine penicillin G is the first-line treatment and highly effective, there are several factors that may lead to treatment failure and recurrence. These factors include inadequate antibiotic dosing or completion of the course, reinfection, and host factors such as immunosuppression [18]. In this patient with chronic liver cirrhosis, the associated altered immunity may have played a part in inadequate treatment or reinfection of *Treponema pallidum*.

Public health importance

Due to its ability to mimic various disease presentations and severe potential complications, the re-emergence of syphilis remains a primary public health concern. Although there was a prior decline in the 2000s, the World Health Organization (WHO) reported an incidence of eight million cases among adults aged 15-49 years in 2022 [20]. Public health initiatives should focus not only on appropriate screening and diagnosis but also on patient education and prevention.

## Conclusions

Primary care physicians, dermatologists, and other healthcare providers should maintain a high index of suspicion when evaluating patients who present with rash and systemic symptoms that mimic preexisting conditions or are not explained by alternative diagnoses. Given the recent resurgence of syphilis and the severity of its potential complications, timely diagnosis and appropriate treatment are imperative. Routine screening should be implemented in all high-risk populations, and established follow-up guidelines must be strictly followed to ensure adequate treatment and disease resolution. The possibility of a prozone effect must be integrated into the differential diagnosis of an atypical serological profile to prevent significant delay in treatment.
